# Binary and Non-binary Gender Identities, Internalizing Problems, and Treatment Wishes Among Adolescents Referred to a Gender Identity Clinic in Germany

**DOI:** 10.1007/s10508-023-02674-8

**Published:** 2023-08-10

**Authors:** Lena Herrmann, Claus Barkmann, Carola Bindt, Saskia Fahrenkrug, Franziska Breu, Jörn Grebe, Inga Becker-Hebly

**Affiliations:** Department of Child and Adolescent Psychiatry, Psychotherapy, and Psychosomatics, University Medical Center Hamburg-Eppendorf, Martinistraße 52, W29, 20246 Hamburg, Germany

**Keywords:** Non-binary, Transgender, Gender dysphoria, Internalizing problems

## Abstract

Clinical research on transgender and gender-nonconforming (TGNC) adolescents has focused on binary individuals or often not differentiated among gender identities. Recent studies suggest that a considerable proportion of TGNC adolescents identify as non-binary and that these youth report more internalizing problems as well as different transition-related medical treatment wishes than binary adolescents. However, the results are inconclusive, and data for the German-speaking area are lacking. Therefore, the present study aimed to assess the percentage of binary and non-binary gender identities in a German sample of clinically referred TGNC adolescents and examine associations of gender identity with internalizing problems and transition-related medical treatment wishes. The sample consisted of 369 adolescents (11–18 years, *M*_age_ = 15.43; 305 birth-assigned female, 64 birth-assigned male) who attended the Hamburg Gender Identity Service for children and adolescents (Hamburg GIS) between 2013 and 2019. Gender identity and treatment wishes were assessed using study-specific items and internalizing problems using the Youth Self-Report. In total, 90% (*n* = 332) of the sample identified as binary and 10% (*n* = 37) as non-binary. Having a non-binary gender identity was significantly associated with more internalizing problems and with wishing for no transition-related medical treatment or only puberty-suppressing hormones. The results underscore that non-binary adolescents represent a specifically vulnerable subgroup within TGNC adolescents with unique mental health needs and treatment wishes. Future research should differentiate among various gender identities. In clinical practice, it is crucial to create an inclusive space for non-binary youth and provide mental health care if needed.

**Supplementary Information:**

The online version contains supplementary material available at 10.1007/s10508-023-02674-8.

## Introduction

Most clinical research on transgender and gender-nonconforming (TGNC) adolescents or youth with a gender dysphoria (GD) diagnosis focused on comparing TGNC with cisgender individuals or subgroups of young TGNC individuals according to their birth-assigned sex (Turban & Ehrensaft, 2018). However, there are more subgroups within TGNC youth with unique experiences and needs, and the experiences of TGNC adolescents of the same birth-assigned sex are therefore not always similar. For instance, non-binary youth are an understudied subgroup with different mental health issues and treatment desires who have only recently become increasingly visible in clinical settings as well as in clinical research (Richards et al., 2016).

The term TGNC refers to a broad range of individuals who experience an incongruence between their gender and their birth-assigned sex (gender incongruence; American Psychological Association, 2015). TGNC individuals with a male sex assigned at birth are often referred to as AMAB (assigned male at birth), and those with a female sex assigned at birth are referred to as AFAB (assigned female at birth). Gender dysphoria (GD) describes a clinical diagnosis related to distress that can arise from gender incongruence (American Psychiatric Association, 2013).

Gender identity refers to one’s inherent sense of being a male, female, or an alternative gender (American Psychological Association, 2015). TGNC individuals’ gender identities are heterogeneous, which is increasingly acknowledged (Motmans et al., 2019). The majority of TGNC individuals identify with the “opposite” gender in a binary understanding, meaning that they identify as either female or male or as either transmasculine or transfeminine (Chew et al., 2020; Richards et al., 2016). However, a considerable and increasing proportion of TGNC individuals (see paragraph further below) identify as non-binary (James et al., 2016; Richards et al., 2016). Non-binary individuals identify between, outside, or beyond the gender binary (Thorne et al., 2019). The umbrella term “non-binary” also embraces different identities, such as “genderfluid” (alternating between different genders), “genderqueer” (gender experience between or outside the gender binary), and “agender” (not identifying with any gender or rejecting the idea of genders). Of note, language for and conceptualizations of gender (minority) identities have changed substantially in recent years. Therefore, the above terms are also likely to change, highlighting the need to respect the preferred self-definitions of individuals (Richards et al., 2016).

For the adolescent and adult general population, three population-based studies demonstrated that the experience of gender identity includes more than just two congruent (defined as stronger identification with one’s own birth-assigned sex than the other sex) or incongruent categories (defined as stronger identification with the other sex than with the birth-assigned sex; Becker et al., 2017; Kuyper & Wijsen, 2014; Van Caenegem et al., 2015). Across these studies, various gender experiences (e.g., ambivalent, no clear gender identity/ neither female nor male) were more prevalent in the adolescent and adult general population than binary-incongruent experiences as defined above (Becker et al., 2017; Kuyper & Wijsen, 2014; Van Caenegem et al., 2015). These findings emphasize the need to go beyond the binary understanding of gender identities (i.e., cisgender vs. transgender or transfemale vs. transmale) to capture the whole range of gender identity—also, or especially, among TGNC individuals.

Among TGNC adolescents specifically, percentages of non-binary gender identities vary considerably across studies and study populations (for an overview of studies focusing on binary vs. non-binary youth in clinical and non-clinical studies, see Table S1 in the Supplementary Material). Altogether, in non-clinical surveys, a wide range of approximately 20–70% of TGNC youth (primarily recruited through social media) identify as non-binary (e.g., Atteberry-Ash et al., 2021; Clark et al., 2018; McKay & Watson, 2020; Roberts et al., 2021; Thoma et al., 2019; Table S1). Since not all and therefore probably fewer non-binary than binary TGNC youth attend specialized gender identity services, the proportion of non-binary youth in clinical studies is lower: Clinical studies indicate that 6–26% of youth attending specialized gender identity services identify as non-binary (e.g., Cheung et al., 2020; Mirabella et al., 2022; O'Bryan et al., 2018; Thorne et al., 2018; Twist & de Graaf, 2019; Table S1). Handler et al. (2019) reported that AFAB adolescents identified significantly more often as non-binary than AMAB adolescents, whereas three other clinical studies found no sex differences in frequencies of non-binary gender identities (Mirabella et al., 2022; Thorne et al., 2018; Twist & de Graaf, 2019). Interestingly (although not the focus of the present study), in an Italian survey, non-binary adolescents self-reported higher levels of gender fluidity than binary adolescents, meaning that they experienced their gender identity as less stable/fixed and more fluid over time and context (Mirabella et al., 2022).

### Internalizing Psychological Problems in Non-binary Adolescents

The minority stress model theorizes that mental health disparities can be caused by the stress associated with gender minority-related stigma, prejudice, and discrimination (Hendricks & Testa, 2012; Meyer, 2003). Such mental health disparities have been well documented for TGNC youth. Compared to their cisgender counterparts, TGNC adolescents report more psychological difficulties or so-called behavioral and emotional problems, especially internalizing problems and elevated rates of depression, suicidality, self-harm, and eating disorders (Bechard et al., 2017; Connolly et al., 2016; de Graaf et al., 2022; Hartig et al., 2022; Levitan et al., 2019). Non-binary young individuals may face even more discrimination and victimization than binary TGNC individuals because their gender expression (e.g., gender-neutral pronouns and non-binary outward appearance) is more conflicting with the gender binary of either female or male (Lefevor et al., 2019). As a result, non-binary youth may have an even higher risk for mental health issues than binary youth.

Studies focusing on these potential differences between binary and non-binary adolescents, especially clinically referred ones, remain scarce, and their results are inconsistent (Chew et al., 2020; Table S1). In several clinical and non-clinical studies, non-binary youth had more mental health issues than binary TGNC youth (Atteberry-Ash et al., 2021; Ciria-Barreiro et al., 2021; Thorne et al., 2018; Veale et al., 2017; Wang et al., 2020). Across these studies, mental health disparities between binary and non-binary adolescents were most pronounced in internalizing disorders and symptoms such as depression, anxiety, suicidality, and self-harming behavior. Likewise, in a British and Dutch clinical study, identifying more strongly with a non-binary gender identity and being AFAB was associated with more internalizing problems among clinically referred TGNC adolescents (de Graaf et al., 2021).

In contrast, other clinical and non-clinical studies found no evidence for elevated rates of internalizing conditions in non-binary youth compared to binary youth (e.g., Aparicio-García et al., 2018; Fontanari et al., 2020; Rusow et al., 2022; Tordoff et al., 2022; Table S1). Furthermore, some studies highlight that there may be differences between AFAB and AMAB non-binary adolescents, highlighting the need to take the birth-assigned sex into account (McKay & Watson, 2020; Parodi et al., 2022; Rimes et al., 2019; Thoma et al., 2019).

Research has identified several risk and protective factors for behavioral and emotional problems in TGNC adolescents in general. First, difficulties in social interactions with peers (such as bullying) or so-called “poor peer relations” have a significant and negative impact on psychological functioning in young TGNC individuals (de Vries et al., 2016; Levitan et al., 2019; Shiffman et al., 2016; Sievert et al., 2021). Second, family support and general family functioning (or the lack thereof) seem to contribute to better (or worse) psychological outcomes in TGNC children and adolescents (Levitan et al., 2019; Sievert et al., 2021; Simons et al., 2013). Non-binary adolescents might receive even less family support and face even more peer problems/bullying than their binary counterparts (due to a lack of societal understanding and acceptance of non-binary gender identities), which might contribute to the elevated rates of internalizing problems in non-binary adolescents (Aparicio-García et al., 2018). Furthermore, many TGNC adolescents report less body satisfaction or less favorable body image scores than their cisgender peers or norm comparison groups (Becker et al., 2018). Additionally, studies show that body image may contribute to the psychological functioning and quality of life in TGNC adolescents (Röder et al., 2019; Verveen et al., 2023). Researchers have suggested that better mental health outcomes among non-binary than among binary TGNC individuals, as observed in a few studies, may reflect higher levels of body satisfaction and gender congruence (Jones et al., 2019). In conclusion and in addition to gender identity, several other factors (birth-assigned sex, poor peer relations, family functioning, and body satisfaction) are related to mental health outcomes in TGNC adolescents and should therefore be considered in the assessment of their psychological functioning/mental health.

### Transition-Related Medical Treatment Wishes of Non-binary Adolescents

Transition-related medical treatments—that is, puberty-suppressing hormones (gonadotrophin-releasing hormone analogues, GnRHa), gender-affirming hormones, and gender-affirming surgeries—can contribute to better mental health outcomes, more life satisfaction, and increased body satisfaction among TGNC youth (Becker et al., 2018; Becker-Hebly et al., 2021; de Vries et al., 2014; Green et al., 2022; Kuper et al., 2020; van der Miesen et al., 2020). However, non-binary TGNC individuals may not desire or need any or fewer of the transition-related medical treatments mentioned above (so-called partial treatment requests, e.g., requesting gender-affirming hormones but not surgeries) because they may experience lower gender incongruence and less body related dysphoria or more body satisfaction than binary TGNC individuals (Jones et al., 2019).

Little is known about the transition-related medical treatment wishes of non-binary youth, with only a few studies specifically focusing on clinical populations of TGNC youth (Table S1). In most studies, non-binary youth were significantly less likely to wish for transition-related medical treatment than binary youth (Clark et al., 2018; Peng et al., 2019; Todd et al., 2019). For instance, Clark et al. reported that 25% of non-binary youth, compared to 85% of binary youth, stated that gender-affirming hormones were necessary for them.

However, in a recent clinical Italian study, transition-related medical treatment wishes did not significantly differ between non-binary and binary adolescents, although non-binary adolescents tended to wish more often for puberty-suppressing hormones (60% vs. 49%) and less often for genital surgery (53% vs. 67%) than binary adolescents (Mirabella et al., 2022). Several studies demonstrated in addition that young non-binary individuals reported barriers to hormone access (e.g., lack of parental support) more often and were more often undecided regarding their own possible hormone therapy than binary individuals (Clark et al., 2018, 2020; Cohen et al., 2022; Green et al., 2022). Young non-binary TGNC people also hesitated to disclose their non-binary gender identities for fear of not receiving the desired transition-related medical treatment (Carlile et al., 2021). Furthermore, two case studies document that non-binary adolescents may desire treatment with puberty-suppressing but not gender-affirming hormones to achieve a gender-neutral or androgynous appearance (Notini et al., 2020; Pang et al., 2020). In summary, the majority of these studies suggest that non-binary adolescents may desire transition-related medical treatments that achieve an affirmation of the “opposite” gender (according to traditional binary notions of gender) less often than binary adolescents.

### Study Aims and Research Questions

To date, there are no studies assessing the percentage of binary or non-binary gender identities in adolescents attending a German gender identity service. Additionally, there are few clinical studies on the association between non-binary gender identities and internalizing problems in TGNC adolescents, and their results are inconclusive. Furthermore, there is still little knowledge on how many non-binary adolescents wish to undergo transition-related medical treatment and which other factors might influence their treatment wishes. Therefore, the present clinical study focuses on the associations of gender identity with internalizing problems and transition-related medical treatment wishes, aiming to answer the following research questions:

1. What is the percentage of binary and non-binary gender identities in AFAB and AMAB adolescents referred to a specialized gender identity service?
2. How is gender identity (binary vs. non-binary) associated with internalizing problems in clinically referred TGNC adolescents?
3. How is gender identity (binary vs. non-binary) associated with transition-related medical treatment wishes in clinically referred TGNC adolescents?

Based on previous findings, we had two hypotheses. First, we hypothesized that having a non-binary gender identity (as opposed to a binary gender identity) would be associated with different levels of internalizing problems. Second, we hypothesized that having a non-binary gender identity (as opposed to a binary gender identity) would be associated with different transition-related medical treatment wishes.

## Method

### Participants

The present study used a cross-sectional, questionnaire-based study design and assessed a cohort of clinically referred adolescents aged 11–18 years. Data were collected from September 2013 to December 2019 at the Hamburg Gender Identity Service for children and adolescents (Hamburg GIS). The Hamburg GIS at the University Medical Center Hamburg-Eppendorf provides specialized diagnostics, counseling, and treatment for TGNC children and adolescents. Since 2013, all families attending the Hamburg GIS have been invited to participate in the study and to complete a set of self-report and parent-report questionnaires at the time of their first appointment. Hence, voluntary participation took place prior to undergoing any form of counseling or treatment. Ethical approval for this study was granted by the local ethics committee. Written informed consent was obtained from all participating adolescents and their parents/caregivers.

**Fig. 1:**
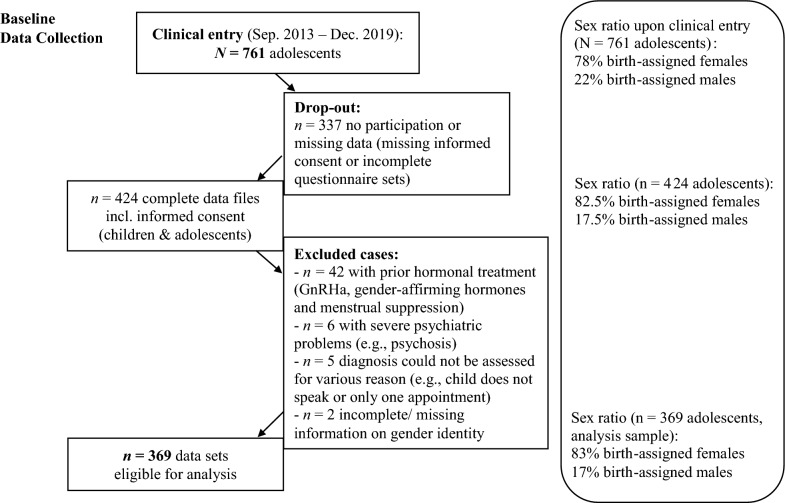
Participants and sex ratios at the Hamburg GIS for children and adolescents

The study population consisted of adolescents (aged 11 years and older) who had been consecutively referred to the Hamburg GIS. During that time, 761 adolescents and their parents/caregivers attended the Hamburg GIS (78% AFAB, 22% AMAB; Fig. 1). In total, complete data sets for 424 participants were available. However, 55 cases had to be excluded for various reasons (Fig. 1). For instance, adolescents with prior hormonal treatment (*n* = 42) were excluded because the study focused on treatment naïve adolescents. Thus, in total, the study sample included 369 adolescents with a diagnosis of GD (aged 11–18 years; 83% AFAB, 17% AMAB).

### Measures

#### Gender Identity

For gender identity, adolescents were asked, “How would you currently describe your gender identity?” They were given the following list of self-identifications and asked to choose the one that fits best: 0 = “female”, 1 = “male”, 2 = “in-between”, 3 = “trans woman/girl”, 4 = “trans man/boy”, and 5 = “other (namely)” (with a write-in option).

Based on their self-identifications, three categories were assigned to the answers: 0 = binary (“female” or “male”), 1 = binary trans (“trans woman/girl” or “trans man/boy”), and 2 = non-binary (“in-between”). Respondents who indicated both binary and binary trans identities (“male” and “trans woman/girl” or “female” and “trans many/boy”) were allocated to the binary trans group. The open answers of the respondents who indicated having an “other” gender identity were screened by the first and last authors and then assigned to one of the three groups (e.g., answers such as “male and trans man/boy” as binary trans and answers such as “agender,” “demi boy,” or “queer and non-binary” as non-binary). If an open answer included both binary trans and non-binary terminologies (e.g., “trans boy” and “non-binary”), the respondent was categorized as non-binary. For analysis purposes, the binary and binary trans groups were later combined, labeled “binary” and compared to the non-binary group. Hence, two gender identity groups were built: 0 = binary and 1 = non-binary. Binary and non-binary adolescents were additionally broken down by their birth-assigned sex to explore possible differences between groups: 0 = AFAB and 1 = AMAB.

#### Sociodemographic Characteristics

The following sociodemographic characteristics were coded: birth-assigned sex, age at assessment (upon clinical entry), citizenship, parental marital status and living situation, and parental socioeconomic status.

The socioeconomic status was assessed using the parent-reported Winkler Index (Winkler & Stolzenberg, 1999). This measure takes parents’ education, income, and job position into account. The Winkler Index has a sum score that ranges from 3 to 21. For this purpose, we used only the following three 3-point variables resulting in a sum score ranging from 3 to 9: educational background of the parent with the highest status (1 = no or lower education, 2 = middle or technical school, 3 = higher education or university), household income (1 = less than 2000 € per month, 2 = 2000–4000 € per month, 3 = more than 4000 € per month), and job position of the parent with the highest status (1 = lower occupation or unemployed, 2 = skilled occupation or self-employed, 3 = executive or academic occupation). The following categories were built: 0 = low socioeconomic status (scores from 3 to 4), 1 = medium socioeconomic status (scores from 5 to 7), and 2 = high socioeconomic status (scores from 8 to 9).

The parents’ marital status and living situation were measured by asking the parents about the current living situation and the relationship status. As suggested by Levitan et al. (2019), two categories were built: 0 = both parents living together or married and 1 = other (living as a single parent, separated, divorced, widowed, or living with a new partner).

#### Internalizing Problems

Internalizing problems were assessed by the German version of the 1991 Youth Self-Report (YSR) (Achenbach, 1991; Döpfner et al., 1998). The YSR is a 119-item self-report questionnaire for adolescents aged 11–18 years. Items are rated on a 3-point scale ranging from 0 (“not true”) to 2 (“very true or often true”) and refer to the past 6 months (“now or within the past 6 months”). *T* scores for the three main scales (total problem score, internalizing, and externalizing problems) were calculated using the German population-based, age- and sex-specific norms provided in Döpfner et al. (1998) to determine whether the scores of the present sample lie within the normal range of the German population. Additionally, clinical range scores (> 90th percentile; *T* scores > 63) were built. Cronbach’s α for the internalizing scale was .91.

For exploratory purposes, we also calculated the YSR externalizing problem and the total problem scores (sum of all problems) to examine psychological functioning more broadly. The following items were excluded from the calculation of the total problem score: asthma (Item 2), allergies (Item 4), socially desirable items (16 items), and cross-gender identification (Item 5 and Item 110). TGNC adolescents might endorse other items on the YSR (than Item 5 and Item 110) for gender-related reasons. Therefore, as described in previous studies (Cohen-Kettenis et al., 2003; de Vries et al., 2016), Item 84 and Item 85 were set to zero if the free-text answers were gender-related to avoid artificial inflation. In our study, Cronbach’s α for the externalizing and the total problem scale were .85 and .94, respectively.

#### Poor Peer Relations

We used the following three items from the YSR to create an index of poor peer relations: Item 25 (“I don’t get along with other kids”), Item 38 (“I get teased a lot”), and Item 48 (“I am not liked by other kids”). The Poor Peer Relation index was developed by Zucker et al. (1997) and has been used in previous studies to measure problematic social interactions with peers of young individuals referred for GD (de Vries et al., 2016; Levitan et al., 2019; Sievert et al., 2021; Zucker et al., 2002, 2012). The index ranges from 0 to 6, with higher scores reflecting poorer peer relations. In the present study, Cronbach’s α was .67.

#### General Family Functioning

General family functioning was assessed using the McMaster Family Assessment Device (FAD; Epstein et al., 1983). The questionnaire has been used in previous studies on TGNC children and adolescents (Levitan et al., 2019; Sievert et al., 2021). The FAD is a questionnaire evaluating family relationships according to the McMaster Model of Family Functioning. For the present study, only the FAD subscale on general family functioning was evaluated. The general family functioning scale consists of 12 items, such as family acceptance (e.g., “Individuals are accepted for what they are” or “We feel accepted for what we are”). The adolescents are asked to decide how well these items describe their own family and to rate the items on a 4-point scale (from 1 = “strongly agree” to 4 = “strongly disagree”). The items are then added and divided by the number of items to determine an average general family functioning score, which can range from 1 to 4, with higher scores indicating lower levels of family functioning. The cutoff for categorical analyses (problematic or unhealthy family functioning) is 2.17 (Byles et al., 1988). Cronbach’s α in the present sample was .88.

#### Suicidality

We used two items from the YSR to create an index for suicidality: Item 18 on self-harming behavior and suicide attempt (“I deliberately try to hurt or kill myself”) and Item 91 on suicidal ideation/thoughts (“I think about killing myself”). The index has been used in various previous studies on TGNC adolescents’ suicidality (de Graaf et al., 2022; Hartig et al., 2022; Van Cauwenberg et al., 2021). The sum score ranges from 0 to 4, with higher scores indicating higher levels of suicidality. Cronbach’s α for the present sample was .75.

#### Body Satisfaction

The pictorial measure Hamburg Body Drawing Scale (HBDS) was used to assess body satisfaction (Appelt & Strauß, 1988; Becker et al., 2016). Participants were given a visualized body figure drawing and asked to rate their satisfaction with 35 body features (e.g., chin, shoulders, and height) and their overall appearance on a 5-point scale (from 1 = “very dissatisfied” to 5 = “very satisfied”). The HBDS has been validated for TGNC populations, and the internal consistency for the HBDS subscales (Cronbach’s α =.63–.91) is satisfactory (Becker et al., 2016). For the present study, only a single item to measure the satisfaction with the overall appearance was used (“satisfaction with the overall appearance”; Becker et al., 2016).

#### Transition-Related Medical Treatment Wishes

To obtain information on transition-related medical treatment wishes, adolescents were asked if they wished to receive the following treatment options in the future: 0 = “puberty-suppressing hormones”, 1 = “gender-affirming hormones”, 2 = “gender-affirming surgical treatment (namely)” (with a write-in option), and 3 = “other (namely)” (with a write-in option). Transition-related medical treatment wishes were then divided into the following two categories based on whether they achieved an affirmation of the “opposite” gender in a binary sense: 0 = “gender-affirming hormones and/or surgical treatments” and 1 = “no treatment wish or only with puberty-suppressing hormones.”

### Statistical Analyses

For the frequency of binary and non-binary gender identities, confidence intervals (95% CI) were calculated. For continuous variables, two-way analyses of variance (ANOVAs) were used to explore differences in the sociodemographic and clinical characteristics between sex (AFAB, AMAB) and genders (binary, non-binary). For categorical variables, exploratory chi-square tests were conducted. Standardized effect sizes (partial eta squared, η_p_^2^ and odds ratios, OR) were calculated to quantify the magnitude of the effect.

Internalizing problems were descriptively evaluated using raw scores, *T* scores, and clinical ranges (> 90th percentile; *T* scores > 63) for the YSR internalizing scale. Additionally, confidence intervals for the *T* scores were reported to compare our study sample with the age- and sex-equivalent population-based German norms (Döpfner et al., 1998). Whenever confidence intervals were not within the normal range of the *T* distribution (*M* = 50, *SD* = 10), a significant difference from the reference group can be assumed. If confidence intervals overlap, the results do not significantly differ from each other (Cumming & Finch, 2005). Although the present study focused on internalizing problems, given the current state of the literature, the YSR externalizing scale and the total problem score were also evaluated for exploratory purposes.

A multiple linear regression analysis was performed to evaluate our first hypothesis on the association between gender identity and internalizing problems. The raw scores of the YSR internalizing scale were used as an outcome variable while controlling for birth-assigned sex, age, poor peer relations, general family functioning, body satisfaction, and the interaction birth-assigned sex x age. The same was conducted to explore the relationships of gender identity with externalizing problems and the total problem score. For the total problem score, three items on poor peer relations (Items 25, 38, and 48) were excluded since the poor peer relation index was a separate predictor in the model. An a priori power analysis (using G*Power) demonstrated that in a multiple regression analysis with 369 cases and seven predictors, a small effect (*f* = 0.02) could be tested with a power of 80%.

For testing our second hypothesis, a multiple logistic regression analysis was conducted to study the association between gender identity and medical treatment wishes while controlling for birth-assigned sex, age, and body satisfaction. A power analysis showed that in a logistic regression analysis with 369 cases, an observed effect of OR = 1.45 could be detected with a power of 80%.

Single missing values were imputed by using the expectation maximization algorithm (Little & Rubin, 2019). All statistical analyses were performed using SPSS 27.

## Results

### Percentage of Binary and Non-binary Gender Identities

In total, 90% (95% CI = 86.4, 92.8; *n* = 332) of the clinically referred adolescents identified as binary. In the binary group, 46% identified as binary (either male or female) and 54% as binary trans (either trans woman/girl or trans man/boy).^1^

Among the 369 adolescents, 37 adolescents identified as non-binary. Thus, the percentage of non-binary gender identities in the present sample was 10% (95% CI = 7.2, 13.6). Most non-binary adolescents (76%) identified with a gender between female and male.^2^ There was a significant association between birth-assigned sex and gender identity (χ^2^(1, 369) = 6.53, *p* = .011, OR = 2.59): AMAB adolescents reported a non-binary gender identity (19%) significantly more often than AFAB adolescents (8%). Conversely, AFAB adolescents identified significantly more often as binary (92%) than AMAB adolescents (81%).

### Sociodemographic and Clinical Characteristics

Table 1 presents more details on the sociodemographic and clinical characteristics of the sample. The TGNC adolescents (*n* = 369) consisted of 83% AFAB and 17% AMAB individuals with a mean age of 15.4 years. There were no age differences between groups.

**Table 1:** Sociodemographic and clinical characteristics as a function of (birth-assigned) sex and gender (identity)

	AFAB (*n* = 305)		AMAB (*n* = 64)		Total (*n* = 369)		Sex comparison				Gender comparison				Interaction (sex × gender)			
	*M*	*SD*	*M*	*SD*	*M*	*SD*	*F*	*df*	*p*	η_p_^2^	*F*	*df*	*p*	η_p_^2^	*F*	*df*	*p*	η_p_^2^
*Age at assessment (in years)*																		
Binary	15.44	1.49	15.48	1.64	15.45	1.51												
Non-binary	15.22	1.95	15.49	1.82	15.31	1.89												
Total	15.42	1.53	15.48	1.66	15.43	1.55	0.28	1 (365)	.596	.00	0.12	1 (365)	.726	*.*00	0.16	1 (365)	.688	.00
*Parental socioeconomic status (Winkler Index)*																		
Binary	6.46	1.64	6.40	1.71	6.45	1.65												
Non-binary	6.64	1.63	6.92	1.51	6.73	1.57												
Total	6.48	1.64	6.50	1.67	6.48	1.64	0.12	1 (365)	.732	.00	1.20	1 (365)	.275	.00	0.29	1 (365)	.593	.00
*Poor peer relations (YSR)*																		
Binary	1.45	1.39	1.94	1.53	1.53	1.42												
Non-binary	1.72	1.70	2.33	1.50	1.92	1.64												
Total	1.48	1.41	2.02	1.52	1.57	1.45	4.05	1 (365)	.045	.01	1.44	1 (365)	.231	.00	0.05	1 (365)	.820	.00
*General family functioning (FAD)*																		
Binary	1.95	0.60	1.95	0.56	1.95	0.59												
Non-binary	1.76	0.47	1.81	0.42	1.77	0.45												
Total	1.94	0.59	1.92	0.53	1.93	0.58	0.04	1 (365)	.846	.00	2.28	1 (365)	.132	.01	0.05	1 (365)	.828	.00
*Suicidality index (YSR)*																		
Binary	1.16	1.34	0.69	1.21	1.09	1.33												
Non-binary	1.72	1.49	0.50	0.80	1.32	1.42												
Total	1.21	1.36	0.66	1.14	1.11	1.34	11.22	1 (365)	< .001	.03	0.53	1 (365)	.467	.00	2.22	1 (365)	.137	.01
*Body satisfaction (HBDS)*																		
Binary	2.34	0.84	2.50	0.86	2.36	0.85												
Non-binary	2.61	0.91	2.57	0.62	2.60	0.82												
Total	2.36	0.85	2.51	0.81	2.39	0.85	0.15	1 (365)	.700	.00	1.18	1 (365)	.277	.00	0.40	1 (365)	.526	.00

The Winkler Index ranges from 3 to 9 (9 = highest socioeconomic status), the poor peer relations sum score from 0 to 6 (6 = worst peer relations), the FAD from 1 to 4 (4 = lowest levels of family functioning), the suicidality index from 0 to 4 (4 = highest levels of suicidality), and the HBDS from 1 to 5 (5 = most body satisfaction)

*AFAB/AMAB* assigned female/male at birth, *FAD* McMaster Family Assessment Device, *HBDS* Hamburg Body Drawing Scale, *YSR* Youth Self-Report

Most adolescents were German citizens (96%) and came from a family with a medium (57%) or high (30%) socioeconomic background. Parents were still living together or married for half of the adolescents (50%). More than two-thirds of the adolescents reported having encountered at least one peer-related problem in the past six months on the poor peer relations scale. Family interactions measured via the general family functioning scale were on average unproblematic (below the cutoff at 2.17). One-third reported problematic family functioning (above the cutoff at 2.17). AMAB adolescents reported on average significantly more peer-related problems than AFAB adolescents. There were no other significant sex or gender differences for any of these variables.

Almost half of the adolescents (45%) reported that they sometimes or often harmed themselves or attempted suicide. Furthermore, 34% of the adolescents endorsed that they sometimes or often had suicidal thoughts. AFAB adolescents reported, on average, significantly higher levels of suicidality than AMAB adolescents. There were no significant differences between binary and non-binary adolescents.

Body satisfaction was on average low. Both non-binary and binary and AFAB and AMAB TGNC adolescents reported that they were, on average, unsatisfied with their overall appearance, with a tendency of more body satisfaction in non-binary and AMAB adolescents. There were no significant sex or gender differences.

### Internalizing Problems

The results for internalizing problems are shown in Table 2. Compared to the German norm population (*M* = 50, *SD* = 10), TGNC adolescents (binary and non-binary as well as AFAB and AMAB) had significantly higher *T* scores (95% CI not including *M* = 50) for internalizing problems. TGNC adolescents scored on average more than 1.5 *SD* higher on the internalizing problem scale than same-aged and same-sex adolescents from the YSR reference group. More than half of the TGNC adolescents reported internalizing problems within the clinical range (> 90th percentile; *T* scores > 63). Non-binary and AMAB adolescents reported even more internalizing problems (*T* scores 2 *SD* above *M* = 50): 70% of non-binary and AMAB adolescents scored within the clinical range of internalizing problems, compared to 55% of binary and 54% of AFAB adolescents. However, the groups did not differ significantly (overlapping 95% CI).

**Table 2:** Internalizing problems (YSR) in AFAB and AMAB binary and non-binary adolescents compared to the German norm population

	Raw scores (TGNC adolescents)			*T* scores (TGNC adolescents with reference to norms)			Clinical range (*T* scores > 63)	
	*M*	*SD*	95% CI	*M*	*SD*	95% CI	%	*n*
*AFAB*								
Binary	21.43	10.68	[20.18, 22.69]	65.24	10.68	[63.98, 66.50]	52.9	148
Non-binary	25.44	12.58	[20.25, 30.63]	69.16	13.17	[63.73, 74.59]	68.0	17
Total	21.76	10.88	[20.53, 22.99]	65.56	10.93	[64.33, 66.79]	54.1	165
*AMAB*								
Binary	20.33	9.52	[17.68, 22.98]	68.31	10.60	[65.36, 71.26]	69.2	36
Non-binary	21.50	7.47	[16.76, 26.24]	69.75	7.82	[64.78, 74.72]	75.0	9
Total	20.55	9.13	[18.27, 22.83]	68.58	10.10	[66.06, 71.10]	70.3	45
*Total*								
Binary	21.26	10.50	[20.13, 22.39]	65.72	10.71	[64.56, 66.88]	55.4	184
Non-binary	24.16	11.22	[20.42, 27.90]	69.35	11.59	[65.49, 73.22]	70.3	26
Total	21.55	10.60	[20.47, 22.64]	66.08	10.84	[64.97, 67.19]	56.9	210

Age and birth-assigned sex equivalent German norms were derived from Döpfner et al. (1998). If confidence intervals do not include the mean of the norm *T* distribution (*M* = 50), a significant deviation from the reference group (adolescents from the general population) can be assumed. Clinical scores (*T* > 63) indicate that 89% of the non-referred age- and sex-equivalent reference group had a lower internalizing problem score. Raw scores for the internalizing scale range from 0 to 62, and *T* scores range from 25 to 100

*AFAB/AMAB* assigned female/male at birth, *TGNC* transgender and gender-nonconforming, *YSR* Youth Self-Report

The results of the multiple linear regression analysis are shown in Table 3. The regression analysis showed that a female birth-assigned sex, poorer peer relations, lower levels of family functioning, less body satisfaction, and having a non-binary gender identity were associated with more internalizing problems. The final model explained 40% of the variance in internalizing problems, while the control variables in total explained 39% and gender identity explained 1%.

**Table 3:** Multiple linear regression analysis: Association between gender identity and internalizing problems (YSR)

	*b*	*SE b*	*ß*	*p*
Intercept	12.92***	2.17		< .001
Birth-assigned sex (0 = assigned female at birth, 1 = assigned male at birth)	− 2.65*	1.16	− .10	.023
Age in years (centered)	0.36	0.32	.05	.252
Interaction [birth-assigned sex x age]	0.05	0.70	.00	.949
Poor peer relations (YSR)	2.75***	0.32	.38	< .001
General family functioning (FAD)	5.55***	0.80	.30	< .001
Body satisfaction (HBDS)	− 2.66***	0.53	− .21	< .001
Gender identity (0 = binary, 1 = non-binary)	3.94**	1.45	.11	.007

Results of the final model: *F*(7, 361) = 35.74, adjusted *R*^*2*^ = .40,* p* < .001

*FAD* McMaster Family Assessment Device, *HBDS* Hamburg Body Drawing Scale, *YSR* Youth Self-Report

*p < .05, **p < .01, ***p < .001

### Transition-Related Medical Treatment Wishes

In total, 91% of TGNC adolescents wished to be treated with gender affirmation (hormones and/or surgeries). Conversely, only 9% wished for no transition-related medical treatment at all or only puberty-suppressing treatment with hormones (GnRHa). The two gender identity groups differed significantly in their transition-related medical treatment wishes (χ^2^(1, 369) = 44.17, *p* <.001, OR = 10.62): compared to 95% of binary adolescents, only 62% of non-binary adolescents wished for treatment with gender-affirming hormones and/or surgeries. Conversely, 38% of the non-binary sample compared to 5% of the binary sample wished for no treatment or only puberty-suppressing treatment. The odds of wishing for no transition-related medical treatment or only puberty-suppressing treatment were 10.6 times higher in non-binary adolescents than in binary adolescents.

Table 4 provides an overview of the multiple logistic regression analysis and the results of the final model. Birth-assigned sex, age, and gender identity were significantly associated with transition-related medical treatment wishes, meaning that the odds of wishing for no transition-related medical treatment at all or only puberty-suppressing treatment were higher among adolescents who were AMAB, younger, and non-binary. After controlling for birth-assigned sex, age, and body satisfaction, non-binary adolescents had 9.8 times higher odds of wishing for no transition-related medical treatment or only puberty-suppressing treatment than binary adolescents. The model explained 30% of the variance in transition-related medical treatment wishes, while all control variables together explained 18% and gender identity explained 12%.

**Table 4:** Multiple logistic regression analysis: Association between gender identity and medical treatment wishes (0 = gender affirming hormones and/or surgical treatments, 1 = no treatment wish or only with puberty-suppressing hormones)

	*B*	*SE*	OR	95% CI for OR	*p*
Intercept	2.02	2.14	7.57		.344
Birth-assigned sex (0 = assigned female at birth, 1 = assigned male at birth)	1.42	0.44	4.13**	[1.75, 9.78]	.001
Age in years	− 0.41	0.13	0.67**	[0.52, 0.86]	.002
Body satisfaction (HBDS)	0.33	0.26	1.39	[0.84, 2.29]	.201
Gender identity (0 = binary, 1 = non-binary)	2.28	0.46	9.77***	[3.95, 24.15]	< .001

Results of the final model: χ^2^(4) = 53.37, *p* < .001, *R*^*2*^ (Nagelkerke) = .30

*HBDS* Hamburg Body Drawing Scale

**p < .01, ***p < .001

### Exploratory Data Analyses

Additional exploratory data analyses were conducted to assess the associations among gender identity with externalizing problems and the total problem score (Supplementary Material). Bivariate analyses showed that externalizing problems were less common than internalizing problems but were still elevated in TGNC adolescents (*T* scores 0.5 *SD* above *M* = 50) (Table S2). In total, 16% of the sample reported clinically relevant externalizing problems. AFAB adolescents reported, on average, significantly more externalizing problems than AMAB adolescents (non-overlapping 95% CI). There were no significant differences between binary and non-binary adolescents. The total problem score was also elevated: TGNC adolescents scored more than 1 *SD* higher than the reference group and 45% fell within the clinical range. Non-binary adolescents tended to have higher total problem scores and to score more often within the clinical range than binary adolescents, but the differences were not significant.

In the multiple regression analysis for externalizing problems, significant predictors were birth-assigned sex, poor peer relations, and general family functioning (Table S3). Gender identity was not associated with externalizing problems. The seven factors together explained 15% of the variance.

For the total problem score, birth-assigned sex, poor peer relations, general family functioning, and body satisfaction were significant predictors, whereas gender identity was not (Table S4). The final model explained 38% of the variance for the total problem score.

## Discussion

The current study aimed to build on previous research by examining the percentage of binary and non-binary gender identities in clinically referred TGNC adolescents attending a German gender identity service. In addition, the present study intended to explore the associations between these two gender identities and internalizing problems and transition-related medical treatment wishes.

In total, 90% of the clinical sample of TGNC adolescents identified as binary and 10% as non-binary. These numbers are consistent with others reported from international gender identity services (non-binary = 6–26%; e.g., Cheung et al., 2020; Mirabella et al., 2022; O'Bryan et al., 2018; Thorne et al., 2018; Twist & de Graaf, 2019). In contrast to other services, however, the percentage of non-binary gender identities was significantly higher among AMAB adolescents (19%) than among AFAB adolescents (8%). However, the sex difference should be interpreted with caution because of the small non-binary sample size (*n* = 37), especially among AMAB adolescents (*n* = 12), and the extreme proportion of referred AFAB individuals. Moreover, the sex ratio and the percentage of non-binary identities among TGNC adolescents may be different today than at the time of data collection (2013–2019), as TGNC individuals increasingly appear to identify as non-binary (James et al., 2016). For instance, in a subsequent (unpublished) study from the Hamburg GIS assessing more recent data (2020–2021), 17% of TGNC adolescents reported having a non-binary gender identity or were still questioning their gender identity. However, most of these adolescents, particularly AMAB adolescents, did not identify as non-binary but were still questioning their gender identity (Herrmann et al., 2023).

Although the effect was small, having a non-binary gender identity was significantly associated with more internalizing problems. Hence, our result is in line with several studies documenting impaired mental health in non-binary youth (Atteberry-Ash et al., 2021; Ciria-Barreiro et al., 2021; Thorne et al., 2018; Veale et al., 2017; Wang et al., 2020). The present study cannot answer for how long the internalizing problems have existed and if they resulted from experienced minority stress or if they may have existed before the onset of the adolescents’ gender dysphoria. However, the minority stress model suggests that both binary and non-binary TGNC individuals frequently experience stigmatization and discrimination, which can result in mental health disparities (Hendricks & Testa, 2012; Meyer, 2003). Compared to binary individuals, non-binary individuals may encounter even more stigmatization and discrimination because they may not conform to the societal expectation of presenting in a binary way (i.e., as a woman or man) or may not pass as a binary gender (Lefevor et al., 2019). Furthermore, experiences such as being repeatedly misgendered may contribute to the feeling of being invisible or not validated by others (de Graaf et al., 2021). As a consequence, non-binary individuals may hide their gender identity because they fear negative reactions or a lack of understanding (Lefevor et al., 2019).

Additionally, intrinsic factors also may be relevant; for instance, having a gender identity that is not often represented (e.g., lacking role models) and informed about (e.g., lacking resources and information) can lead to a long-lasting search for or struggling with one’s gender identity (de Graaf et al., 2021; Thorne et al., 2018). As a result, the process of gender identity exploration may be more stressful and confusing for young non-binary individuals (de Graaf et al., 2021) and may also influence their presentation during counseling or when they wish to undergo medical treatment. Non-binary individuals also may have fewer protective factors, such as self-esteem or good peer relations (de Graaf et al., 2021; Thorne et al., 2018). Additionally, in a few cases, a non-binary identification could theoretically also reflect fears of growing up or intrapsychic conflicts (e.g., reluctance to identify as binary trans or homosexual) (Notini et al., 2020; Pang et al., 2020). In conclusion, non-binary youth may have even greater mental health needs than binary TGNC youth, emphasizing the importance of providing corresponding mental health care or counseling for this vulnerable subgroup.

Our exploratory analyses revealed that gender identity was not associated with externalizing problems and the YSR total problem score, emphasizing that particularly internalizing problems, such as depression, anxiety, and suicidal ideation, seem to be elevated in non-binary adolescents specifically (Atteberry-Ash et al., 2021; Thorne et al., 2018; Veale et al., 2017; Wang et al., 2020). Of note, both binary and non-binary adolescents reported significantly more problems on all YSR scales (internalizing, externalizing, and total problem scores) compared to adolescents from the general population, but internalizing problems were especially elevated. Concerning suicidality, nearly half of TGNC adolescents reported self-harming behavior or suicide attempts, and one-third had suicidal ideation/thoughts. Unfortunately, we could not differentiate between self-harming behavior and suicide attempts because the YSR assesses both behaviors with one item, which might also explain the contra intuitive higher prevalence of self-harm and suicide attempts (probably, in most cases, rather self-harm than suicide attempts) than of suicidal ideation/thoughts. When comparing our YSR data to those of other international gender identity clinics, the present sample scored similarly on all YSR scales and reported similar levels of peer-related problems and suicidality (de Graaf et al., 2018, 2022).

In accordance with most previous studies (Clark et al., 2018; Peng et al., 2019; Todd et al., 2019), identifying as non-binary was significantly associated with wishing for no gender-affirming treatment. Non-binary youth may feel more comfortable with living in their gender without needing gender-affirming treatment, for example, because they are more comfortable with an androgynous appearance or because they do not want to acquire female or male sex characteristics (Clark et al., 2018; Notini et al., 2020). On the other hand, they may be less informed or undecided about possible treatment options (Clark et al., 2018, 2020; Cohen et al., 2022). These untraditional treatment pathways may raise new ethical dilemmas. For instance, long-term puberty suppression, as a way of achieving a gender-neutral/androgynous appearance, is associated with increased health risks (e.g., reduced bone density, impaired fertility and sexual functioning) and may not be completely reversible (Notini et al., 2020). A strong evidence base for such untraditional treatment decisions is absent, which makes decisions in these cases even more complex, especially for young individuals (Notini et al., 2020; Pang et al., 2020). Given that young individuals are in the middle of their development and gender might be even more fluid in non-binary than in binary adolescents (Mirabella et al., 2022), the assessment and decision-making process might be more time-consuming and ethically complex. Assisting the achievement of a more non-binary appearance also may additionally lead to increased stigmatization by others, highlighting the need to discuss such risks with adolescents (Notini et al., 2020).

### Limitations

The present findings should be interpreted in light of some limitations. First, “non-binary” was not an exclusive/specific option on the list of gender self-identifications that the participants could choose, since the term was not so common when the study was developed, and the fear was to overwhelm youth with such terms. Instead, non-binary participants could choose the option “in-between” (which is nowadays an outdated term) or write in their gender identity as a free-text answer. However, only three participants specifically described themselves clearly as “non-binary.” This is not only a limitation of the present study but also underscores that “non-binary” is merely an emerging umbrella term that tries to comprise different gender identities outside the binary (Thorne et al., 2019). As a result, the constantly evolving language for gender minority identities can make research in this field more challenging and requires a continuous adaption of measures. Working with community experts and allowing participants to write in their preferred terms (e.g., for gender identities or pronouns) is therefore recommended.

Second, all participants attended a specialized gender identity service. This way, we could examine an underresearched topic in a clinical sample. It is, however, possible that many non-binary adolescents do not seek specialized care because they do not wish for any kind of transition-related medical treatment. Therefore, our clinical sample is probably not representative of the possibly diverse non-binary population. Since the study was conducted in the same clinic, in which the adolescents also were receiving support/treatment, they may have hesitated to disclose their (non-binary) gender identity or to participate in the study due to fear of barriers to access of care (Carlile et al., 2021). In Germany, transition-related medical interventions are currently neither recommended (or only in very few cases), nor is it foreseen that insurance covers their costs for non-binary adolescents (Arbeitsgemeinschaft der Wissenschaftlichen Medizinischen Fachgesellschaften e. V. (AWMF), 2013; Medizinischer Dienst des Spitzenverbandes Bund der Krankenkassen (MDS), 2020). Additionally, most TGNC adolescents were AFAB, thus, further limiting the representativeness and diversity of the sample and our findings. Since the sex ratio was also very unbalanced at clinical entry (Figure 1), this reflects less of a problem of drop-out or adherence but rather underlines that internationally predominantly AFAB adolescents present to gender identity clinics. Although the reasons for the imbalanced sex ratio in TGNC adolescents are unknown, various experts have suggested that, for example, a different age of puberty onset (with an on average earlier onset in AFAB youth) might lead to differential developmental pathways (Aitken et al., 2015; de Graaf et al., 2018).

Third, although compared to other clinical studies, the total sample was relatively large (*n* = 369), the non-binary subsample was relatively small (*n* = 37). This difference in subsample sizes is not surprising since non-binary adolescents present a minority group within clinically referred TGNC adolescents. As a result, the sample sizes (binary vs. non-binary) for our hypothesis-testing analyses (i.e., multiple regression analyses) were unbalanced, but still sufficient (approximately 10%). However, the descriptive and exploratory testing results (e.g., externalizing symptoms) should be interpreted cautiously (e.g., estimates of 95% CIs are less reliable in small samples).

Fourth, we applied a cross-sectional study design. Hence, it is not possible to draw any long-term conclusions from the results. Since the participating adolescents completed questionnaires upon clinical entry, before receiving any information, support, or treatment, the transition-related medical treatment wishes of some TGNC adolescents were not “final” or informed. Therefore, the transition-related medical treatment wishes of some TGNC adolescents may have or probably will have changed in the course of their treatment at the Hamburg GIS. It is also possible that the gender identities of some TGNC adolescents will change during the treatment, highlighting the need for longitudinal studies.

### Future Directions and Clinical Implications

Future studies examining the mental health outcomes of clinically referred non-binary adolescents may benefit from investigating more diverse TGNC adolescent populations. A better understanding of intersecting identities (e.g., sexual, religious, and racial) in non-binary adolescents is needed. There also is a need for more longitudinal studies, which can overcome some of the mentioned limitations and provide additional insights into the long-term mental health outcomes of non-binary TGNC adolescents. Moreover, future research would benefit from investigating why non-binary adolescents report more internalizing problems than binary adolescents and how they can be best supported.

Our study provides additional evidence that clinically referred non-binary TGNC adolescents represent a vulnerable group with unique mental health and treatment needs. Both specialized gender identity services and individual clinicians are encouraged to provide an inclusive setting for non-binary youth to ensure that they seek support when needed. An open, non-binary-inclusive setting and mindset will probably make it easier for non-binary adolescents to explore and disclose their gender identity.

The present results demonstrated that there was still a considerable proportion of non-binary adolescents who wanted to undergo gender-affirming medical treatment. In light of the evidence that also non-binary youth benefit from receiving transition-related medical treatment (Green et al., 2022) and that the inability to access transition-related medical treatments when desired contributes to decreased well-being (Burgwal et al., 2019), giving access to care to this population is essential. Therefore, clinicians should provide information on the full range of transition-related medical treatment available, without making any assumptions, to ensure that non-binary youth can make informed treatment decisions.

### Conclusions

In the present study, a small but not negligible proportion of TGNC adolescents identified as non-binary. Furthermore, having a non-binary gender identity was significantly associated with more internalizing problems and wishing for no gender-affirming treatment. Hence, the present findings underscore the growing diversity of treatment-seeking TGNC adolescents. As a result, individualized and patient-centered treatment plans become increasingly important to provide the best possible care for a heterogeneous and vulnerable group of young individuals.

### Supplementary Information

Below is the link to the electronic supplementary material.

Supplementary file1 (DOCX 67 kb)

## Data Availability

The data are not publicly available.

## Footnotes

1 Closer examination of the binary group revealed that 45% of the AFAB adolescents identified as male and 55% as trans men/boys. Among the AMAB adolescents, 52% identified as female and 48% as trans women/girls.

2 “Other” non-binary gender identities, given as free-text answers (24%), were “queer or non-binary” (*n* = 1), “demi boy” (*n* = 1), “I am myself “ (*n* = 1), “transgender” (*n* = 2), “trans boy/non-binary” (*n* = 1), “transgender non-binary guy” (*n* = 1), “rather nothing” (*n* = 1), and “neither female nor male, ‘it’” (*n* = 1).
