# Supplementary material for: Binary and Non-binary Gender Identities, Internalizing Problems, and Treatment Wishes Among Adolescents Referred to a Gender Identity Clinic in Germany
Source: Arch Sex Behav. 2023 Aug 10;53(1):91–106. doi: 10.1007/s10508-023-02674-8 (PMC10794330; doi:10.1007/s10508-023-02674-8)
Supplement: Supplementary file 1 — Supplementary file1 (DOCX 67 kb) [file 10508_2023_2674_MOESM1_ESM.docx]

**SUPPLEMENTARY MATERIAL**

| **Table S1** Studies in binary vs. non-binary TGNC youth focusing on the percentage of gender identities, mental health problems, and transition-related medical treatment wishes | | | | | | |
| --- | --- | --- | --- | --- | --- | --- |
| **Authors and year of publication** | **Study type** | **Country** | **Participants** | **Population** | **Focus** | **Main results (for this paper)^1^** |
| Andrzejewski et al., 2021 | Cross-sectional survey | USA | 1,029 TGNC youth (13–24 years; *M*_age_ = 19.1) | Non-clinical | Access to transition-related medical treatment, disease prevention | 26% (*n* = 264) identified as non-binary and 74% (*n* = 765) as binary; non-binary youth had less often accessed transition-related medical services than binary youth (10% vs. 22%) |
| Aparicio-García, 2018 | Cross-sectional survey | Spain | 250 TGNC youth (14–25 years; *M*_age_ = 20.4) and 532 cisgender youth | Non-clinical | Mental health, substance use, risk vs. protective factors | Among TGNC youth, 28% (*n* = 70) identified as non-binary and 72% (*n* = 180) as binary; non-binary and binary youth reported similarly often suicidal thoughts (78% and 70%) and psychological health problems (56% and 52%) |
| Atteberry-Ash et al., 2021 | Cross-sectional survey | USA | 396 TGNC youth (14–18 years) | Non-clinical | Depression, self-injury, suicidal ideation, suicide attempts | 19% (*n* = 76) identified as non-binary, 39% (*n* = 153) as binary, and 42% (*n* = 167) were unsure/questioning; the likelihood of depression was similar across gender identities, but non-binary youth were most likely to report suicide attempts (adjusted OR = 3.42) |
| Avila et al., 2019 | Cross-sectional survey | USA | 106 TGNC youth (13–22 years; *M*_age_ = 16.5) | Clinical | Weight manipulation, disordered eating, hormonal treatment | 11% (*n* = 12) identified as non-binary and 89% (*n* = 94) as binary; non-binary youth were less often on transition-related hormonal treatment than binary youth (17% vs. 32%), but there were no differences between groups regarding weight manipulation and disordered eating behaviors |
| Bosse & Chiodo, 2016 | Cross-sectional survey | USA | 72 TGNC and 103 LGBQ youth (18–25 years; *M*_age_ = 21.1): 78% AFAB, 22% AMAB | Non-clinical | Sexual and gender identity in LGBTQ youth | Among TGNC youth, 55% (*n* = 35) of AFAB youth identified as non-binary and 45% (*n* = 22) as binary, compared to AMAB youth, of whom 50% (*n* = 4) identified as non-binary and 50% (*n* = 8) as binary |
| Boyer et al., 2022 | Cross-sectional survey | USA | 156 TGNC youth (12–26 years) | Clinical | Demographics, non-affirming healthcare experiences | 20% (*n* = 31) identified as non-binary and 80% (*n* = 125) as binary; non-affirming healthcare experiences were sig. more often reported by non-binary (β =1.41) and transmasculine youth (β = 0.78) than by transfeminine youth |
| Carlile et al., 2021 | Interview study | UK | 14 TGNC youth (5 – 25; *M*_age_ = 15.0) and their parents | Clinical | Treatment experiences | Non-binary youth (*n* = 4) were careful to disclose their gender identity for fear of not receiving gender-affirming medical treatment |
| Chen et al., 2021 | Cohort study | USA | 411 TGNC children and adolescents (8–16 years; *M*_age_ = 14.9): 61% AFAB, 39% AMAB | Clinical | Demographics, transition-related medical treatment, mental health, gender-specific experiences | 7% (*n* = 18) of AFAB youth identified as non-binary and 93% (*n* = 233) as binary, compared to AMAB youth, of whom 6% (*n* = 10) identified as non-binary and 94% (*n* = 150) as binary *(no comparisons between gender identity groups regarding main outcomes)* |
| Cheung et al., 2020 | Retrospective chart review | Australia | 362 TGNC children and adolescents (4–18 years) | Clinical | Sociodemographics, mental health, treatment history | 8% in the pediatric clinic identified as non-binary and 92% as binary (*n* for pediatric patients not reported) |
| Childs et al., 2022 | Retrospective chart review | USA | 17 TGNC and 409 cisgender adolescents (12–17 years; *M*_age_ = 14.9) | Clinical  (psychiatric outpatient clinic) | Psychological and psychosocial functioning | Among TGNC adolescents, 41% (*n* = 7) identified as non-binary and 59% (*n* = 10) as binary; similar levels of psychological and psychosocial functioning among non-binary and binary TGNC adolescents (e.g., depression score *M* = 75 vs. *M* = 68) |
| Ciria-Barreiro et al., 2021 | Cross-sectional survey | Spain | 303 TGNC and 909 cisgender adolescents (15–18 years) | Non-clinical | Life satisfaction, quality of life, health | Among TGNC adolescents, 70% (*n* = 213) identified as non-binary and 30% (*n* = 90) as binary; non-binary adolescents reported sig. more psychological complaints than binary TGNC adolescents (*d* = 0.55) but did not differ in levels of life satisfaction and quality of life |
| Clark et al., 2018 | Cross-sectional survey | Canada | 839 TGNC youth (14–25 years) | Non-clinical | Sociodemographic characteristics, health issues, health care access | 41% (*n* = 344) identified as non-binary and 49% (*n* = 495) as binary; a sig. larger proportion of AFAB youth was in the non-binary than in the binary group (82% vs. 70%); sig. worse mental health reported by non-binary than binary youth (*M* = 0.84 vs. *M* = 1.02; OR = 0.63); non-binary youth were sig. less likely to have ever taken hormone therapy than binary youth (13% vs. 50%; OR = 0.39) and to report that hormones are necessary for them (25% vs. 85%; OR = 0.05); non-binary youth in comparison to binary youth were sig. more likely to be still deciding about (45% vs. 13%) or not planning hormone therapy (30% vs. 2%); non-binary youth were sig. more likely to report barriers to needed hormone therapy than binary youth (37% vs. 23%; OR = 2.01) |
| Clark et al., 2020 | Interview study | Canada | 21 TGNC adolescents (14–18 years) and their parents | Non-clinical | Treatment decisions. barriers to transition-related medical care | Non-binary adolescents (*n* = 5) in comparison to binary adolescents (*n* = 16) were more often undecided or experienced barriers to care (80% vs. 13%) |
| Cohen et al., 2022 | Mixed methods study | USA | 68 TGNC youth (*M*_age_ = 15.1 years):  57% AFAB, 43% AMAB | Clinical | Sociodemographics, shifts in transition-related medical wishes | 22% (*n* = 15) identified as non-binary and 78% (*n* = 53) as binary; shifts in transition-related medical wishes were sig. more often reported by non-binary than binary youth (60% vs. 21%) |
| de Graaf et al., 2021 | Cross-sectional survey | UK (and Nether-lands) | 572 TGNC adolescents (12–18 years; *M*_age_ = 15.7): 75.5% AFAB, 24.5% AMAB | Clinical | Psychological functioning, non-binary identity (on a spectrum) | No differences between AFAB and AMAB youth in identifying across the non-binary spectrum; having a stronger non-binary gender identity was sig. associated with reporting more psychological problems (β = 0.11−0.19) |
| Fontanari et al., 2020 | Cohort study | Brazil | 350 TGNC youth (16–24 years; *M*_age_ = 18.6) | Non-clinical | Transition-related medical treatment, mental health issues | 33% (*n* = 116) identified as non-binary and 67% (*n* = 234) as binary; no differences between non-binary and binary adolescents in levels of depression and anxiety; non-binary youth had sig. less often undergone hormonal or surgical treatment than binary youth (6% vs. 35%) |
| Green et al., 2022 | Cross-sectional survey | USA | 5,735 TGNC youth (13–24 years; *M*_age_ = 17.6) | Non-clinical | Mental health, receipt of gender-affirming hormone therapy | 44% (*n* = 2504) identified as non-binary and 57% (*n* = 3249) as binary; a sig. larger proportion of those who were non-binary than binary reported wanting gender-affirming hormone therapy but not receiving it (90% vs. 71%) |
| Handler et al., 2019 | Retrospective chart review | USA | 417 TGNC children and adolescents (13–17 years; *Mdn*_age_ = 15): 73% AFAB, 27% AMAB | Clinical | Demographics, reasons for referrals, transition-related medical treatment requests | 13% (*n* = 56) identified as non-binary and 86% (*n* = 359) as binary; majority of non-binary youth was AFAB (79%) *(no comparisons between different gender identities regarding treatment requests)* |
| Linsenmeyer et al., 2021 | Cross-sectional survey | USA | 164 TGNC youth (12–23 years; *M*_age_ = 17.0) | Clinical | Disordered eating and food insecurities | 5% (*n* = 8) identified as non-binary and 95% (*n* = 156) as binary; no sig. differences between non-binary and binary adolescents in disordered eating or food insecurities |
| McCay & Watson, 2020 | Cross-sectional survey | USA | 3,624 TGNC and 7,050 cisgender LGBQ adolescents (13–17 years; *M*_age_ = 15.6); 75% AFAB, 25% AMAB | Non-clinical | Mental health issues, gender identity disclosure | Among TGNC youth, 72% (*n* = 2599) identified as non-binary and 28% (*n* = 1025) as binary; trans boys reported the highest levels of depressive symptoms (*M* = 16.97) and sig. more depressive symptoms than trans girls (*M* = 13.58) and non-binary AFAB (*M* = 16.06) and AMAB adolescents (*M* = 13.43); non-binary AFAB reported sig. more depressive symptoms than non-binary AMAB adolescents |
| Mirabella et al., 2022 | Cross-sectional survey | Italy | 125 TGNC adolescents (11–18 years); 68% AFAB, 32% AMAB | Clinical | Gender identity and gender expression, transition-related medical treatment wishes | 26% (*n* = 32) identified as non-binary and 74% (*n* = 93) as binary, with no differences in frequencies between AFAB and AMAB adolescents; non-binary adolescents reported sig. more fluidity in their gender identity than binary adolescents; no sig. differences in transition-related medical treatment wishes between non-binary and binary adolescents |
| Notini et al., 2020 | Case study | Australia | 1 non-binary AFAB youth (18 years) | Clinical | Transition-related medical treatment wishes | Case of Phoenix, who requests ongoing puberty suppression to prevent the development of their secondary sex characteristics |
| O’Bryan et al., 2018 | Retrospective chart review | USA | 139 TGNC children and adolescents (8–21 years; *M*_age_ = 17.5); 70% AFAB, 30% AMAB | Clinical | Demographics, diagnoses, transition-related medical treatment steps | 6% (*n* = 9) identified as non-binary and 94% (*n* = 130) as binary *(no comparisons between gender identity groups regarding transition-related medical treatment steps)* |
| Pang et al., 2020 | Case study/  ethics round | Australia, UK, USA | 1 non-binary AMAB adolescent (15 years) | Clinical | Transition-related medical treatment wishes | Case of EF, who wishes to continue puberty suppression until they are at least 18 years |
| Parodi et al., 2022 | Longitudinal study (baseline data) | USA | 252 TGNC adolescents (14–18 years): 54% AFAB, 46% AMAB | Non-clinical | Anxiety, depression, nonsuicidal self-injury, school-related factors | 52% (*n* = 129) identified as non-binary and 48% (*n* = 123) as binary, with no differences in frequencies between AFAB and AMAB adolescents; non-binary AFAB youth reported sig. more depressive symptoms (*M* = 3.7) than trans girls (*M* = 2.6), but similar levels as trans boys (*M* = 3.2); no differences in levels of anxiety or frequencies of nonsuicidal self-injury between gender identity groups |
| Peng et al., 2019 | Cross-sectional survey | China | 385 TGNC adolescents (12–18 years; *M*_age_ = 16.7) | Non-clinical | Mental health, transition-related medical treatment wishes, abuse, neglect, bullying | 28% (*n* = 109) identified as non-binary and 72% (*n* = 276) as binary; trans girls reported sig. higher levels of mental health problems and suicidal behaviors (*M* = 15.7) than trans boys (*M* = 14.2) and non-binary adolescents (*M* = 14.3); binary adolescents wished sig. more often for hormonal treatment (OR = 4.7–10.7) than non-binary adolescents |
| Pham et al., 2022 | Longitudinal study | USA | 91 TGNC adolescents (8–22 years; *M*_age_ = 15.2 years): 66% AFAB, 34% AMAB | Clinical | Disordered eating | 7% (*n* = 6) identified as non-binary and 93% (*n* = 85) identified as binary; no differences in disordered eating between gender identity groups |
| Poquiz et al., 2020 | Retrospective chart review | USA | 638 TGNC youth (12–24 years; *M*_age_ = 16.1): 73% AFAB, 27% AMAB | Clinical | Demographics, minority stress and resilience | 10% (*n* = 63) identified as non-binary and 90% (*n* = 575) as binary; binary youth reported sig. more discrimination than non-binary youth |
| Price-Feeney et al., 2020 | Cross-sectional survey | USA | 8,381 TGNC and 17,015 LGBQ cisgender youth (13–24 years; *M*_age_ = 18.0): 59% AFAB, 41% AMAB | Non-clinical | Demographics, depressive mood, suicidal ideation, suicide attempts in the past 12 months | Among TGNC youth, 58% (*n* = 4825) identified as non-binary, and 42% (*n* = 3555) as binary; trans boys reported sign. higher rates of depressive mood (86%), suicidal ideation (62%), and suicide attempts (35%) than trans girls (76%, 52%, and 27%) and non-binary adolescents (72%, 43%, and 23%) |
| Rimes et al., 2019 | Cross-sectional survey | UK | 677 TGNC youth (16–25 years; *M*_age_ = 19.9): 71% AFAB, 29% AMAB | Non-clinical | Demographics, mental health, substance use, victimization | 53% (*n* = 362) identified as non-binary and 47% (*n* = 315) as binary; non-binary youth reported similarly high rates of mental health problems (22–48%) as binary youth (28–44%); non-binary AMAB youth reported sig. less often suicide attempts (12%) than binary (30–32%) and AFAB non-binary youth (24%) |
| Roberts et al., 2021 | Cross-sectional study | USA | 1,191 TGNC and 919 cisgender adolescents (14–18 years; *M*_age_ = 15.9): 84% AFAB, 16% AMAB | Non-clinical | Demographics, disordered eating | Among TGNC adolescents, 41% (*n* = 492) identified as non-binary or were questioning their gender identity, and 59% (*n* = 699) were binary; sig. differences between binary and non-binary adolescents regarding muscle building, but not purging, excessive exercise, or caloric restriction |
| Rusow et al., 2022 | Cross-sectional survey | USA | 108 TGNC youth (16–24 years; *M*_age_ = 21.4): 79% AFAB, 21% AMAB | Non-clinical | Mental health, sexual health behaviors, substance use, healthcare utilization | 65% (*n* = 70) identified as non-binary and 35% (*n* = 38) as binary; no differences between binary and non-binary youth regarding depressive or anxiety symptoms |
| Sood et al., 2021 | Cross-sectional survey | USA | 156 transmasculine and non-binary AFAB adolescents (12–18 years; *M*_age_ = 15.3) | Clinical | Chest dysphoria, anxiety, depression, wish for top surgery | 15% (*n* = 24) identified as non-binary or were questioning their gender identity, and 85% (*n* = 132) identified as binary; non-binary/questioning and binary adolescents had similar levels of chest dysphoria *(no comparisons between gender identity groups regarding anxiety and depression)* |
| Thoma et al., 2019 | Cross-sectional survey | USA | 1148 TGNC and 872 cisgender adolescents (14–18 years; *M*_age_ = 15.9) | Non-clinical | Sociodemographics, suicidality, non-suicidal self-injury | Among TGNC adolescents, 41% (*n* = 469) identified as non-binary or were questioning their gender identity, and 59% (*n* = 679) identified as binary; trans boys (89%) and non-binary AFAB adolescents (88%) reported sig. more often non-suicidal self-injury than trans girls (73%) |
| Thorne et al., 2018 | Longitudinal study (baseline findings) | UK | 388 TGNC youth (16–25 years; *M*_age_ = 20.2): 55% AFAB, 45% AMAB | Clinical | Mental health and social support | 15% (*n* = 57) identified as non-binary and 85% (*n* = 331) as binary, with no differences in frequencies between AFAB and AMAB adolescents; non-binary youth reported sig. higher levels of anxiety (*M* = 11.77) and depression (*M* = 9.00) than binary group (*M* = 9.71, *M* = 7.06) |
| Todd et al., 2019 | Longitudinal study (baseline findings) | USA | 202 TGNC youth (15–24 years): 76% AFAB, 24% AMAB | Non-clinical | Sociodemographics, transition-related medical treatment, sexual behaviors, substance use, resilience, stress | 41% (*n* = 83) identified as non-binary and 59% (*n* = 119) as binary; binary youth were sig. more often AFAB than non-binary youth (82% vs. 68%); binary youth were sig. more likely to have accessed transition-related medical care (46% vs. 8%) or plan to do so in the future than non-binary youth (50% vs. 43%) |
| Toomey et al., 2018 | Cross-sectional survey | USA | 1,773 TGNC and 118,844 cisgender adolescents (11–19 years; *M*_age_ = 14.7) | Non-clinical | Demographics, lifetime suicide behavior | Among TGNC adolescents, 19% (*n* = 344) identified as non-binary, 59% (*n* = 1052) were questioning their gender identity, and 21% (*n* = 377) identified as binary; trans boys and non-binary adolescents reported sig. higher rates of lifetime suicide behaviors (51%, 42%) than trans girls (30%) and questioning adolescents (28%) |
| Tordoff et al., 2022 | Longitudinal study | USA | 104 youth (13–20 years; *M*_age_ = 15.8) | Clinical | Depression, anxiety, self-harm, or suicidal thoughts | 10% (*n* = 10) identified as non-binary, 4% (*n* = 4) were unsure or reported no gender identity, and 87% (*n* = 90) identified as binary; no differences in baseline mental health issues between non-binary and binary youth |
| Twist & de Graaf, 2019 | Pilot study | UK | 251 TGNC adolescents (12–17 years; *M*_age_ = 15.4): 71% AFAB, 29% AMAB | Clinical | Gender diversity | 12% identified as non-binary (*n* = 30) and 86% (*n* = 212) as binary (*n* = 72) (2%, *n* = 4 “undefined”), with no differences in frequencies between AFAB and AMAB adolescents |
| Veale et al., 2017 | Cross-sectional survey | Canada | 839 TGNC youth (14–25 years) | Non-clinical | Mental health, gender identity | 41% (*n* = 344) identified as non-binary and 49% (*n* = 495) as binary (see also Clark et al., 2018); non-binary youth reported sig. worse general mental health and more often self-harm than (mostly AFAB) binary youth |
| Van Donge et al., 2019 | Retrospective chart review | USA | 53 TGNC youth (*M*_age_ = 14.5 years): 76% AFAB, 25% AMAB | Clinical (military health care) | Demographics, psychosocial and behavioral risk profile, healthcare utilization | 15% (*n* = 6) identified as non-binary and 85% (*n* = 45) as binary, with no differences in frequencies between AFAB and AMAB adolescents |
| Wang et al., 2020 | Cross-sectional survey | China | 2,111 TGNC and 9,997 cisgender adolescents (grades 7–11; *M*_age_ = 15.8 years): 46% AFAB, 54% AMAB | Non-clinical | Sociodemographics, mental health issues, bullying at school | Among TGNC adolescents, 12% (*n* = 250) identified as non-binary, 38% (*n* = 792) were questioning their gender identity, and 51% (*n* = 1069) identified as binary; non-binary AMAB adolescents had the highest risk of having a suicide plan (OR = 5.4 with cisgender boys as reference), but similar risk for self-harm and suicidal ideation |
| Watson et al., 2019 | Cross-sectional survey | USA | 5,637 TGNC and 11,475 cisgender LGBQ adolescents (13–17 years; *M*_age_ = 15.6): 72% AFAB, 28% AMAB | Non-clinical | Gender and sexual identity labels | Among TGNC adolescents, 72% (*n* = 4048) identified as non-binary and 28% (*n* = 1589) as binary |
| Studies were found via a systematic literature search in Medline on 27 January 2023. The search string is described below.  ^1^Only results relevant to the focus of the paper were summarized. The table does, for example, not present differences between TGNC and cisgender youth.  AFAB/AMAB = assigned female/male at birth, LGBTQ = lesbian, gay, bisexual, transgender, or queer, TGNC = transgender and non-conforming | | | | | | |

**Search string** (by Chew et al., 2020; adapted)

("non-binary"[Title/Abstract] OR "nonbinary"[Title/Abstract] OR "genderqueer"[Title/Abstract] OR "gender-nonconformity"[Title/Abstract] OR "genderfluid"[Title/Abstract] OR "agender"[Title/Abstract] OR "bigender"[Title/Abstract] OR "genderneutral"[Title/Abstract] OR "genderless"[Title/Abstract] OR "non-gendered"[Title/Abstract] OR "third-gender"[Title/Abstract]) AND ("adolescen*"[Title/Abstract] OR "pediatric*"[Title/Abstract] OR "paediatric*"[Title/Abstract] OR "youth*"[Title/Abstract] OR "teen"[Title/Abstract] OR "teens"[Title/Abstract] OR "teenage*"[Title/Abstract])

**References (for the included studies above)**

Andrzejewski, J., Dunville, R., Johns, M. M., Michaels, S., & Reisner, S. L. (2021). Medical gender affirmation and HIV and sexually transmitted disease prevention in transgender youth: Results from the Survey of Today's Adolescent Relationships and Transitions, 2018. *LGBT Health*, *8*(3), 181−189. https://doi.org/10.1089/lgbt.2020.0367

Aparicio-García, M. E., Díaz-Ramiro, E. M., Rubio-Valdehita, S., López-Núñez, M. I., & García-Nieto, I. (2018). Health and well-being of cisgender, transgender and non-binary young people. *International Journal of Environmental Research and Public Health*, *15*(10), Article 2133. https://doi.org/10.3390/ijerph15102133

Atteberry-Ash, B., Kattari, S. K., Harner, V., Prince, D. M., Verdino, A. P., Kattari, L., & Park, I. Y. (2021). Differential experiences of mental health among transgender and gender-diverse youth in Colorado. *Behavioral Sciences*, *11*(4), Article 48. https://doi.org/10.3390/bs11040048

Avila, J. T., Golden, N. H., & Aye, T. (2019). Eating disorder screening in transgender youth. *Journal of Adolescent Health*, *65*(6), 815−817. https://doi.org/10.1016/j.jadohealth.2019.06.011

Bosse, J. D., & Chiodo, L. (2016). It is complicated: Gender and sexual orientation identity in LGBTQ youth. *Journal of Clinical Nursing*, *25*(23−24), 3665−3675. https://doi.org/10.1111/jocn.13419

Boyer, T. L., Sequeira, G. M., Egan, J. E., Ray, K. N., Miller, E., & Coulter, R. W. (2022). Binary and nonbinary transgender adolescents' healthcare experiences, avoidance, and well visits. *Journal of Adolescent Health*, *71*(4), 438−445. https://doi.org/10.1016/j.jadohealth.2022.04.016

Carlile, A., Butteriss, E., & Sansfaçon, A. P. (2021). “It’s like my kid came back overnight”: Experiences of trans and non-binary young people and their families seeking, finding and engaging with clinical care in England. *International Journal of Transgender Health*, *22*(4), 412−424. https://doi.org/10.1080/26895269.2020.1870188

Chen, D., Abrams, M., Clark, L., Ehrensaft, D., Tishelman, A. C., Chan, Y. M., Garofalo, R., Olson-Kennedy, J., Rosenthal, S. M., & Hidalgo, M. A. (2021). Psychosocial characteristics of transgender youth seeking gender-affirming medical treatment: Baseline findings from the Trans Youth Care Study. *Journal of Adolescent Health*, *68*(6), 1104−1111. https://doi.org/10.1016/j.jadohealth.2020.07.033

Cheung, A. S., Leemaqz, S. Y., Wong, J. W., Chew, D., Ooi, O., Cundill, P., Silberstein, N., Locke, P., Zwickl, S., Grayon, R., Zajac, J. D., & Pang, K. C. (2020). Non-binary and binary gender identity in Australian trans and gender diverse individuals. *Archives of Sexual Behavior*, *49*, 2673−2681. https://doi.org/10.1007/s10508-020-01689-9

Childs, A. W., Kaufman, C. C., & Olezeski, C. L. (2022). How is everyone doing? Baseline psychological distress and adaptive functioning among transgender, nonbinary, and cis youth presenting for intensive outpatient psychiatric services. *Psychological Services*, *19*(3), 541−550. https://doi.org/10.1037/ser0000573

Ciria-Barreiro, E., Moreno-Maldonado, C., Rivera, F., & Moreno, C. (2021). A comparative study of health and well-being among cisgender and binary and nonbinary transgender adolescents in Spain. *LGBT Health*, *8*(8), 536−544. https://doi.org/10.1089/lgbt.2020.0477

Clark, B. A., Veale, J. F., Townsend, M., Frohard-Dourlent, H., & Saewyc, E. (2018). Non-binary youth: Access to gender-affirming primary health care. *International Journal of Transgenderism, 19*(2), 158−169. https://doi.org/10.1080/15532739.2017.1394954

Clark, B. A., Marshall, S. K., & Saewyc, E. M. (2020). Hormone therapy decision-making processes: Transgender youth and parents. *Journal of Adolescence*, *79*, 136−147. https://doi.org/10.1016/j.adolescence.2019.12.016

Cohen, A., Gomez-Lobo, V., Willing, L., Call, D., Damle, L. F., D'Angelo, L. J., Song, A., & Strang, J. F. (2022). Shifts in gender-related medical requests by transgender and gender-diverse adolescents. *Journal of Adolescent Health*, *72*(3), 428–436. https://doi.org/10.1016/j.jadohealth.2022.10.020

de Graaf, N. M., Huisman, B., Cohen-Kettenis, P. T., Twist, J., Hage, K., Carmichael, P., Kreukels, B. P. C., & Steensma, T. D. (2021). Psychological functioning in non-binary identifying adolescents and adults. *Journal of Sex and Marital Therapy*, *47*(8), 773−784. https://doi.org/10.1080/0092623X.2021.1950087

Fontanari, A. M. V., Vilanova, F., Schneider, M. A., Chinazzo, I., Soll, B. M., Schwarz, K., Lobato, M. I. R. & Brandelli Costa, A. (2020). Gender affirmation is associated with transgender and gender nonbinary youth mental health improvement. *LGBT Health*, *7*(5), 237−247. https://doi.org/10.1089/lgbt.2019.0046

Green, A. E., DeChants, J. P., Price, M. N., & Davis, C. K. (2022). Association of gender-affirming hormone therapy with depression, thoughts of suicide, and attempted suicide among transgender and nonbinary youth. *Journal of Adolescent Health*, *70*(4), 643−649. https://doi.org/10.1016/j.jadohealth.2021.10.036

Handler, T., Hojilla, J., Varghese, R., Wellenstein, W., Satre, D. D., & Zaritsky, E. (2019). Trends in referrals to a pediatric transgender clinic. *Pediatrics*, *144*(5), Article e20191368. https://doi.org/10.1542/peds.2019-1368

Linsenmeyer, W. R., Katz, I. M., Reed, J. L., Giedinghagen, A. M., Lewis, C. B., & Garwood, S. K. (2021). Disordered eating, food insecurity, and weight status among transgender and gender nonbinary youth and young adults: A cross-sectional study using a nutrition screening protocol. *LGBT Health*, *8*(5), 359−366. https://doi.org/10.1089/lgbt.2020.0308

McKay, T. R., & Watson, R. J. (2020). Gender expansive youth disclosure and mental health: Clinical implications of gender identity disclosure. *Psychology of Sexual Orientation and Gender Diversity*, *7*(1), 66−75. https://doi.org/10.1037/sgd0000354

Mirabella, M., Piras, I., Fortunato, A., Fisher, A. D., Lingiardi, V., Mosconi, M., Ristori, J., Speranza, A. M., & Giovanardi, G. (2022). Gender identity and non-binary presentations in adolescents attending two specialized services in Italy. *Journal of Sexual Medicine*, *19*(6), 1035−1048. https://doi.org/10.1016/j.jsxm.2022.03.215

Notini, L., Earp, B. D., Gillam, L., McDougall, R. J., Savulescu, J., Telfer, M., & Pang, K. C. (2020). Forever young? The ethics of ongoing puberty suppression for non-binary adults. *Journal of Medical Ethics*, *46*(11), 743−752. https://doi.org/10.1136/medethics-2019-106012

O'Bryan, J., Leon, K., Wolf-Gould, C., Scribani, M., Tallman, N., & Gadomski, A. (2018). Building a pediatric patient registry to study health outcomes among transgender and gender expansive youth at a rural gender clinic. *Transgender Health*, *3*(1), 179−189. https://doi.org/10.1089/trgh.2018.0023

Pang, K. C., Notini, L., McDougall, R., Gillam, L., Savulescu, J., Wilkinson, D., Clark, B. A., Olson-Kennedy, J., Telfer, M. M., & Lantos, J. D. (2020). Long-term puberty suppression for a nonbinary teenager. *Pediatrics*, *145*(2), Article e20191606. https://doi.org/10.1542/peds.2019-1606

Parodi, K. B., Holt, M. K., Green, J. G., Katz-Wise, S. L., Shah, T. N., Kraus, A. D., & Xuan, Z. (2022). Associations between school-related factors and mental health among transgender and gender diverse youth. *Journal of School Psychology*, *90*, 135−149. https://doi.org/10.1016/j.jsp.2021.11.004

Peng, K., Zhu, X., Gillespie, A., Wang, Y., Gao, Y., Xin, Y., Ou, J., Zhong, S., Zhao, L., Liu, J., Wang, C., & Chen, R. (2019). Self-reported rates of abuse, neglect, and bullying experienced by transgender and gender-nonbinary adolescents in China. *JAMA Network Open*, *2*(9), Article e1911058. https://doi.org/10.1001/jamanetworkopen.2019.11058

Pham, A. H., Eadeh, H. M., Garrison, M. M., & Ahrens, K. R. (2022). A longitudinal study on disordered eating in transgender and non-binary adolescents. *Academic Pediatrics*. Advance online publication. https://doi.org/10.1016/j.acap.2022.12.013

Poquiz, J. L., Coyne, C. A., Garofalo, R., & Chen, D. (2021). Comparison of gender minority stress and resilience among transmasculine, transfeminine, and nonbinary adolescents and young adults. *Journal of Adolescent Health*, *68*(3), 615−618. https://doi.org/10.1016/j.jadohealth.2020.06.014

Price-Feeney, M., Green, A. E., & Dorison, S. (2020). Understanding the mental health of transgender and nonbinary youth. *Journal of Adolescent Health*, *66*(6), 684−690. https://doi.org/10.1016/j.jadohealth.2019.11.314

Rimes, K. A., Goodship, N., Ussher, G., Baker, D., & West, E. (2019). Non-binary and binary transgender youth: Comparison of mental health, self-harm, suicidality, substance use and victimization experiences. *International Journal of Transgenderism*, *20*(2−3), 230−240. https://doi.org/10.1080/15532739.2017.1370627

Roberts, S. R., Salk, R. H., Thoma, B. C., Romito, M., Levine, M. D., & Choukas‐Bradley, S. (2021). Disparities in disordered eating between gender minority and cisgender adolescents. *International Journal of Eating Disorders*, *54*(7), 1135−1146. https://doi.org/10.1002/eat.23494

Rusow, J. A., Hidalgo, M. A., Calvetti, S., Quint, M., Wu, S., Bray, B. C., & Kipke, M. D. (2022). Health and service utilization among a sample of gender-diverse youth of color: the TRUTH study. *BMC Public Health*, *22*(1), Article 2312. https://doi.org/10.1186/s12889-022-14585-9

Sood, R., Chen, D., Muldoon, A. L., Chen, L., Kwasny, M. J., Simons, L. K., Gangopadhyay, N., Corcoran, J. F., & Jordan, S. W. (2021). Association of chest dysphoria with anxiety and depression in transmasculine and nonbinary adolescents seeking gender-affirming care. *Journal of Adolescent Health*, *68*(6), 1135−1141. https://doi.org/10.1016/j.jadohealth.2021.02.024

Thoma, B. C., Salk, R. H., Choukas-Bradley, S., Goldstein, T. R., Levine, M. D., & Marshal, M. P. (2019). Suicidality disparities between transgender and cisgender adolescents. *Pediatrics*, *144*(5), Article e20191183. https://doi.org/10.1542/peds.2019-1183

Thorne, N., Witcomb, G. L., Nieder, T., Nixon, E., Yip, A., & Arcelus, J. (2019). A comparison of mental health symptomatology and levels of social support in young treatment seeking transgender individuals who identify as binary and non-binary. *International Journal of Transgenderism*, *20*(2−3), 241−250. https://doi.org/10.1080/15532739.2018.1452660

Todd, K., Peitzmeier, S. M., Kattari, S. K., Miller-Perusse, M., Sharma, A., & Stephenson, R. (2019). Demographic and behavioral profiles of nonbinary and binary transgender youth. *Transgender Health*, *4*(1), 254−261. https://doi.org/10.1089/trgh.2018.0068

Toomey, R. B., Syvertsen, A. K., & Shramko, M. (2018). Transgender adolescent suicide behavior. *Pediatrics*, *142*(4), Article e20174218. https://doi.org/10.1542/peds.2017-4218

Tordoff, D. M., Wanta, J. W., Collin, A., Stepney, C., Inwards-Breland, D. J., & Ahrens, K. (2022). Mental health outcomes in transgender and nonbinary youths receiving gender-affirming care. *JAMA Network Open*, *5*(2), Article e220978. https://doi.org/10.1001/jamanetworkopen.2022.0978

Twist, J., & de Graaf, N. M. (2019). Gender diversity and non-binary presentations in young people attending the United Kingdom’s National Gender Identity Development Service. *Clinical Child Psychology and Psychiatry*, *24*(2), 277−290. https://doi.org/10.1177/1359104518804311

Veale, J. F., Watson, R. J., Peter, T., & Saewyc, E. M. (2017). Mental health disparities among Canadian transgender youth. *Journal of Adolescent Health, 60*(1), 44−49. https://doi.org/10.1016/j.jadohealth.2016.09.014

Van Donge, N., Schvey, N. A., Roberts, T. A., & Klein, D. A. (2019). Transgender dependent adolescents in the US military health care system: Demographics, treatments sought, and health care service utilization. *Military Medicine*, *184*(5−6), e447−e454. https://doi.org/10.1093/milmed/usy264

Wang, Y., Yu, H., Yang, Y., Drescher, J., Li, R., Yin, W., Yu, R., Wang, S., Deng, W., Qiufang, J., Zucker, K. J., & Chen, R. (2020). Mental health status of cisgender and gender-diverse secondary school students in China. *JAMA Network Open*, *3*(10), Article e2022796. https://doi.org/10.1001/jamanetworkopen.2020.22796

Watson, R. J., Wheldon, C. W., & Puhl, R. M. (2020). Evidence of diverse identities in a large national sample of sexual and gender minority adolescents. *Journal of Research on Adolescence*, *30*, 431−442. https://doi.org/10.1111/jora.12488

| **Table S2** Exploratory analysis of externalizing problems and the total problem score (YSR) in AFAB and AMAB non-binary and binary identifying adolescents compared to the German norm population | | | | | | | | | | |
| --- | --- | --- | --- | --- | --- | --- | --- | --- | --- | --- |
|  | **Raw scores** (TGNC adolescents) | | |  | ***T* scores** (TGNC adolescents with reference to norms) | | |  | **Clinical range** (*T* scores > 63) | |
|  | *M* | *SD* | 95% CI |  | *M* | *SD* | 95% CI |  | % | *n* |
| ***Externalizing scale*** |  |  |  |  |  |  |  |  |  |  |
| **AFAB** |  |  |  |  |  |  |  |  |  |  |
| Binary | 13.04 | 6.97 | [12.22, 13.86] |  | 56.16 | 8.15 | [55.20, 57.12] |  | 18.2 | 51 |
| Non-binary | 11.88 | 8.70 | [8.29, 15.47] |  | 54.12 | 10.84 | [49.65, 58.59] |  | 16.0 | 4 |
| Total | 12.94 | 7.12 | [12.14, 13.74] |  | 55.99 | 8.40 | [55.05, 56.94] |  | 18.0 | 55 |
| **AMAB** |  |  |  |  |  |  |  |  |  |  |
| Binary | 10.77 | 5.51 | [9.23, 12.30] |  | 52.83 | 6.42 | [51.04, 54.61] |  | 5.8 | 3 |
| Non-binary | 11.25 | 9.38 | [5.29, 17.21] |  | 53.08 | 10.01 | [46.72, 59.45] |  | 8.3 | 1 |
| Total | 10.86 | 6.32 | [9.28, 12.44] |  | 52.88 | 7.13 | [51.09, 54.66] |  | 6.3 | 4 |
| **Total** |  |  |  |  |  |  |  |  |  |  |
| Binary | 12.68 | 6.80 | [11.95, 13.42] |  | 55.64 | 7.98 | [54.78, 56.50] |  | 16.3 | 54 |
| Non-binary | 11.68 | 8.80 | [8.74, 14.61] |  | 53.78 | 10.45 | [50.30, 57.27] |  | 13.5 | 5 |
| Total | 12.58 | 7.02 | [11.86, 13.30] |  | 55.45 | 8.27 | [54.61, 56.30] |  | 16.0 | 59 |
| ***Total problem score*** |  |  |  |  |  |  |  |  |  |  |
| **AFAB** |  |  |  |  |  |  |  |  |  |  |
| Binary | 54.49 | 23.67 | [51.71, 57.28] |  | 63.04 | 9.30 | [61.95, 64.14] |  | 45.0 | 126 |
| Non-binary | 58.44 | 26.56 | [47.48, 69.40] |  | 64.24 | 10.73 | [59.81, 68.67] |  | 52.0 | 13 |
| Total | 54.82 | 23.89 | [52.12, 57.51] |  | 63.14 | 9.41 | [62.08, 64.20] |  | 45.6 | 139 |
| **AMAB** |  |  |  |  |  |  |  |  |  |  |
| Binary | 50.52 | 20.03 | [44.94, 56.10] |  | 62.62 | 7.83 | [60.43, 64.80] |  | 42.3 | 22 |
| Non-binary | 54.00 | 17.79 | [42.70, 65.30] |  | 64.25 | 6.90 | [59.86, 68.64] |  | 50.0 | 6 |
| Total | 51.17 | 19.54 | [46.29, 56.05] |  | 62.92 | 7.64 | [61.01, 64.83] |  | 43.8 | 28 |
| **Total** |  |  |  |  |  |  |  |  |  |  |
| Binary | 53.87 | 23.15 | [51.37, 56.37] |  | 62.98 | 9.07 | [62.00, 63.96] |  | 44.6 | 148 |
| Non-binary | 57.00 | 23.91 | [49.03, 64.97] |  | 64.24 | 9.56 | [61.06, 67.43] |  | 51.4 | 19 |
| Total | 54.18 | 23.21 | [51.81, 56.56] |  | 63.10 | 9.12 | [62.17, 64.04] |  | 45.3 | 167 |
| Age and sex equivalent German norms were derived from Döpfner et al. (1998). If confidence intervals do not include the mean of the norm *T* distribution (*M* = 50), a significant deviation from the reference group (adolescents from the general population) can be assumed. Clinical scores (*T* > 63) indicate that 89% of the non-referred reference group had a lower externalizing/total problem score. Raw scores for the externalizing scale range from 0 to 60, raw scores for the Total problem score range from 0 to 198, and *T* scores range from 25 to 100. AFAB/AMAB = assigned female/male at birth, TGNC = transgender and gender-nonconforming, YSR = Youth Self-Report | | | | | | | | | | |

| **Table S3** Exploratory multiple linear regression analysis: Association between gender identity and externalizing problems (YSR) | | | | |
| --- | --- | --- | --- | --- |
|  | *b* | *SE b* | *ß* | *p* |
| Intercept | 5.57** | 1.71 |  | .001 |
| Birth-assigned sex (0 = assigned female at birth, 1 = assigned male at birth) | − 2.39** | 0.91 | − .13 | .009 |
| Age in years (centered) | − 0.07 | 0.25 | − .02 | .782 |
| Interaction [birth-assigned sex x age] | − 0.76 | 0.55 | − .08 | .168 |
| Poor peer relations (YSR) | 0.85*** | 0.25 | .18 | <.001 |
| General family functioning (FAD) | 3.47*** | 0.63 | .29 | <.001 |
| Body satisfaction (HBDS) | − 0.24 | 0.42 | − .03 | .562 |
| Gender identity (0 = binary, 1 = non-binary) | − 0.27 | 1.15 | − .01 | .816 |
| Results of the final model: *F*(7, 361) = 10.24, adjusted *R^2^* = .15, *p* <.001  ***p* < .01, ****p* < .001*,* HBDS = Hamburg Body Drawing Scale, FAD = McMasters’ Family Assessment Device, YSR = Youth Self-Report | | | | |

| **Table S4** Exploratory multiple linear regression analysis: Association between gender identity and the total problem score (YSR) | | | | |
| --- | --- | --- | --- | --- |
|  | *b* | *SE b* | *ß* | *p* |
| Intercept | 27.47*** | 4.68 |  | <.001 |
| Birth-assigned sex (0 = assigned female at birth, 1 = assigned male at birth) | − 6.75** | 2.50 | − .11 | .007 |
| Age in years (centered) | 0.36 | 0.68 | .03 | .596 |
| Interaction [birth-assigned sex x age] | − 1.57 | 1.51 | − .05 | .298 |
| Poor peer relations (YSR) | 5.34*** | 0.69 | .34 | <.001 |
| General family functioning (FAD) | 14.03*** | 1.72 | .36 | <.001 |
| Body satisfaction (HBDS) | − 4.07*** | 1.15 | − .15 | <.001 |
| Gender identity (0 = binary, 1 = non-binary) | 5.31 | 3.14 | .07 | .092 |
| Results of the final model: *F*(7, 361) = 32.92, adjusted *R^2^* = .38, *p* <.001  ***p* < .01, ****p* < .001, HBDS = Hamburg Body Drawing Scale, FAD = McMasters’ Family Assessment Device, YSR = Youth Self-Report | | | | |
